# Editorial: Physiology in Medicine: From Rest to Exercise

**DOI:** 10.3389/fphys.2021.827636

**Published:** 2022-02-08

**Authors:** James P. Fisher, Johannes J. van Lieshout

**Affiliations:** ^1^Department of Physiology, Faculty of Medical and Health Sciences, The University of Auckland, Auckland, New Zealand; ^2^Department of Internal Medicine, University of Amsterdam, Amsterdam, Netherlands; ^3^Laboratory for Clinical Cardiovascular Physiology, Amsterdam University Medical Center, University of Amsterdam, Amsterdam, Netherlands; ^4^Medical Research Council Versus Arthritis Centre for Musculoskeletal Ageing Research, Division of Physiology, Pharmacology and Neuroscience, School of Life Sciences, The Medical School, University of Nottingham Medical School, Queen's Medical Centre, Nottingham, United Kingdom

**Keywords:** oxygen consumption, regulation of skeletal muscle mass, Parkinson's disease and autonomic function, artificial intelligence & hemodynamic monitoring, arterial thrombosis & exercise, atrial fibrillation & cognitive decline, breath-hold diving, T2DM & cerebral perfusion/sympathetic control

In 1993 the Swedish physiologist Björn Folkow (1921–2012) wrote “*.. in humans, it is particularly difficult to differentiate between manifestations of aging per se and symptoms of morbidity, especially at higher ages when morbidity is common*” (Folkow and Svanborg, 1993). In this Research Topic, the articles address some of the difficulties indicated by Folkow in understanding how the human body functions and the diverse effects of healthy aging and disease.

## Featured Publications

Hill and Lupton in 1923 were the first to describe the maximal oxygen uptake (VO_2max_) as “..*the oxygen intake during an exercise intensity at which actual oxygen intake reaches a maximum beyond which no increase in effort can raise it*” (Hill and Lupton, 1923). VO_2max_ is the primary determinant of endurance performance and considered a valid index representing the limits of the cardiorespiratory systems' ability to transport oxygen from the air to the tissues at a given level of physical conditioning and oxygen availability (Hawkins et al., 2007). Martin-Rincon and Calbet demonstrate that the averaging strategy employed to calculate VO_2max_ from breath-by-breath data can change VO_2max_ by 4–10%, and offer practical recommendations for VO_2max_ reporting to permit adequate comparisons in training studies and meta-analyses.

Along with the decline in aerobic capacity, leg extension power, and the number of times that an individual can arise from sit to stand in 30 s starts to decline already at age +50 years (Suetta et al., 2019). Gharahdaghi et al. analyse the importance of hormones like testosterone, estrogen, growth hormone, and insulin-like growth factor in relation to the regulation of skeletal muscle mass and their involvement in the skeletal muscle adaptation to resistance exercise.

The first step to exercise is often the assumption of the upright body position, which itself involves physical activity. The gravitational displacement of blood from the chest to the lower parts of the body elicits a fall in central blood volume and thus cardiac stroke volume. A reduction of the central blood volume in response to postural stress, or immediately after finishing exercise or by blood loss, may be manifest as orthostatic intolerance. A soon as termination of exercise removes the leg muscle pump function the central blood volume is no longer maintained which may provoke post-exercise hypotension. Van der Ster et al. discuss recent developments in artificial intelligence-based monitoring the central blood volume and predicting arterial hypotension during environmental stress ranging from exercise to hemorrhage on the battlefield and in patients during surgery.

In 1817 James Parkinson summarized the shaking palsy or “paralysis agitans” as follows: “..*Involuntary tremulous motion, with lessened muscular power, in parts not in action and even when supported; with a propensity to bend the trunk forwards, and to pass from a walking to a running pace: the senses and intellects being uninjured”* (Parkinson, 2002). Parkinson's disease (PD) is currently the second most common neurodegenerative disorder. In their review, Sabino-Carvalho et al. evaluate the clinically significant and complex associations between autonomic dysfunction, fatigue and exercise capacity in PD.

Human brain function depends on continuous delivery of oxygen and nutrients (Sick et al., 1982) but exhaustive exercise evokes a competition for the supply of oxygenated blood between the brain and the working muscles. Kim et al. allege evidence that an inability to increase cardiac output sufficiently during exercise may jeopardize cerebral perfusion in diabetic patients. The effects of functional activation on the brain by exercise are considered in relation to the regularly limited exercise tolerance in T2D patients experienced as early fatigue. They address the cerebral vs. cardiovascular responses with a focus on the brain's attenuated vascular response to exercise in these patients as compared to healthy subjects.

Exercise training is a well-recognized tool in the management of T2D due to its many cardiometabolic benefits, however T2D may negatively affect cardiovascular responses to exercise. In T2D patients the ability to adjust the circulation during exercise may become impaired, for instance with an augmented blood pressure response but attenuation of blood flow to contracting skeletal muscle. Grotle et al. summarize the current understanding of these altered exercise responses in T2D and the potential underlying mechanisms, with an emphasis on the sympathetic nervous system and its regulation during exercise.

Acute arterial thrombosis is the cause of myocardial infarction and stroke, which in the past decades have collectively become the most common causes of death in the developed countries. Regular physical activity has protective effects on these manifestations of cardiovascular disease, but acute exercise impacts on the coagulation system with a transient increase in the risk of arterial thrombosis. Olsen et al. examine this risk-benefit paradox with regard to physical activity, summarize the experimental methodology presently available to assess the integrated risk of arterial thrombosis and discuss the influence of acute exercise and exercise training on biomarkers of arterial thrombosis.

Atrial fibrillation (AF) is characterized by rapid and irregular beating of the atrial chambers of the heart that increases the risk of cardioembolic events (Naccarelli, 2000). Junejo et al. present evidence for diminished cerebral blood flow and malfunction of its control mechanisms in AF patients and consider the role of cerebrovascular dysfunction as one reason for the heightened risk of cognitive decline, depression, and dementia in AF patients. The associations between exercise and AF are complex. Both low levels of physical activity and very high levels of endurance exercise training appear to increase the risk of developing AF (Flannery et al., 2017), while there is relatively little evidence from randomized controlled trials regarding the benefits and risks of exercise training in adults with AF (Risom et al., 2017).

When studying the diving behavior of Cuvier's beaked whales (*Ziphius cavirostris*) off the Southern California coast using satellite-linked tags the depth record was established in a whale who dived to 2,992 m (9,816ft) without breathing for more than 2 h (Schorr et al., 2014). In contrast, humans are not physiologically and anatomically well-adapted to the environmental stress of diving which involves both exercise and asphyxia during progressive elevations in hydrostatic pressure. Nevertheless, breath-hold diving as another example of environmental stress is an ubiquitous activity. Patrician et al. review the physiology of deep diving focussing on the acute risks of diving but also on the potential long-term medical consequences to breath-hold diving.

## Summary

The issues covered in this Frontier's Clinical and Translational Physiology topic *en passant* illustrate the importance of encouraging crosstalk between physiologists and clinicians who only by joining in research from bench to bedside will get a better understanding how the body functions in health and disease for the benefit of all.

Or in the words of August Steenberg Krogh (1874–1949) awarded the 1920 Nobel Prize in Physiology or Medicine for discovery of the capillary motor-regulating mechanism, when he addressed the International Physiology Congress in 1929 (Krogh, 1929): “.. *I suppose that almost every worker in our science has given some thought to the general progress of physiology and to the problems raised by its growth, and I cannot doubt that some have pondered deeply over these problems and have much more insight into them than I possess, but you must admit that on the whole the thoughts have been kept private, and nobody seems to have considered it worthwhile to bring the matter up for a general discussion among physiologists. When I venture to do so it is because I feel deeply the importance of the subject, and in spite of the fact that I feel even more deeply my own lack of competence in all questions involving organization. My aim is only to draw your attention to some of the problems in the hope that means may be found to solve them*.”

## Author Contributions

JvL and JF wrote and revised the manuscript. All authors contributed to the article and approved the submitted version.

## Funding

JF is supported by the Auckland Medical Research Foundation (1119008), the Health Research Council of New Zealand (19/687) and the Royal Society Te Apārangi (grant number: 19-UOA-170).

## Conflict of Interest

JF has received funding from BMS/Pfizer for an investigator-led and competitively reviewed research project concerning vascular function in atrial fibrillation. The remaining author declares that the research was conducted in the absence of any commercial or financial relationships that could be construed as a potential conflict of interest.

## Publisher's Note

All claims expressed in this article are solely those of the authors and do not necessarily represent those of their affiliated organizations, or those of the publisher, the editors and the reviewers. Any product that may be evaluated in this article, or claim that may be made by its manufacturer, is not guaranteed or endorsed by the publisher.

## References

[B1] Flannery M. D. Kalman J. M. Sanders P. La Gerche A. (2017). State of the art review: atrial fibrillation in athletes. Heart Lung Circul. 26, 983–989. 10.1016/j.hlc.2017.05.132 28606607

[B2] Folkow B. Svanborg A. (1993). Physiology of cardiovascular aging. Physiol. Rev. 73, 725–765. 10.1152/physrev.1993.73.4.725 8105498

[B3] Hawkins M. N. Raven P. B. Snell P. G. Stray-Gundersen J. Levine B. D. (2007). Maximal oxygen uptake as a parametric measure of cardiorespiratory capacity. Med. Sci. Sports Exerc. 39, 103–107. 10.1249/01.mss.0000241641.75101.64 17218891

[B4] Hill A. V. Lupton H. (1923). Muscular exercise, lactic acid, and the supply and utilization of oxygen. QJM Int. J. Med. 16, 135–171. 10.1093/qjmed/os-16.62.135

[B5] Krogh A. (1929). The progress of physiology. Am. J. Physiol. Legacy Content 90, 243–251. 10.1152/ajplegacy.1929.90.2.243

[B6] Naccarelli G. V. (2000). Atrial fibrillation: Edited by Johan Waktare and A. John Camm Martin Dunitz, London (2000) 120 pages, illustrated, $19.95 ISBN: 1-85317-715-6. Clin. Cardiol. 23:310. 10.1002/clc.4960230422

[B7] Parkinson J. (2002). Essay on the shaking palsy. J Neuropsychiatry Clin. Neurosci. 14, 223–236. 10.1176/jnp.14.2.223 11983801

[B8] Risom S. S. Zwisler A. D. Johansen P. P. Sibilitz K. L. Lindschou J. Gluud C.. (2017). Exercise-based cardiac rehabilitation for adults with atrial fibrillation. Cochrane Database Syst. Rev. 2:CD011197. 10.1002/14651858.CD011197.pub2 28181684 PMC6464537

[B9] Schorr G. S. Falcone E. A. Moretti D. J. Andrews R. D. (2014). First long-term behavioral records from Cuvier's beaked whales (Ziphius cavirostris) reveal record-breaking dives. PLoS ONE 9:e92633. 10.1371/journal.pone.0092633 24670984 PMC3966784

[B10] Sick T. J. Lutz P. L. LaManna J. C. Rosenthal M. (1982). Comparative brain oxygenation and mitochondrial redox activity in turtles and rats. J. Appl. Physiol. 53, 1354–1359. 10.1152/jappl.1982.53.6.1354 6295991

[B11] Suetta C. Haddock B. Alcazar J. Noerst T. Hansen O. M. Ludvig H.. (2019). The Copenhagen Sarcopenia Study: lean mass, strength, power, and physical function in a Danish cohort aged 20-93 years. J. Cachexia Sarcopenia Muscle 10, 1316–1329. 10.1002/jcsm.12477 31419087 PMC6903448

